# Comparison of the Impact of Xanthohumol and Phenethyl Isothiocyanate and Their Combination on Nrf2 and NF-κB Pathways in HepG2 Cells In Vitro and Tumor Burden In Vivo

**DOI:** 10.3390/nu13093000

**Published:** 2021-08-28

**Authors:** Marta Cykowiak, Violetta Krajka-Kuźniak, Robert Kleszcz, Małgorzata Kucińska, Hanna Szaefer, Hanna Piotrowska-Kempisty, Adam Plewiński, Marek Murias, Wanda Baer-Dubowska

**Affiliations:** 1 Department of Pharmaceutical Biochemistry, Poznan University of Medical Sciences, 4, Święcickiego Street, 60-781 Poznań, Poland; marta.cykowiak@ump.edu.pl (M.C.); kleszcz@ump.edu.pl (R.K.); hszaefer@ump.edu.pl (H.S.); baerw@ump.edu.pl (W.B.-D.); 2 Department of Toxicology, Poznan University of Medical Sciences, Dojazd 30, 60-631 Poznań, Poland; kucinska@ump.edu.pl (M.K.); hpiotrow@ump.edu.pl (H.P.-K.); marek.murias@ump.edu.pl (M.M.); 3 Centre for Advanced Technologies, Adam Mickiewicz University, 10, Uniwersytetu Poznańskiego Street, 61-614 Poznań, Poland; adam.plewinski@amu.edu.pl

**Keywords:** Nrf2, NF-κB, phytochemicals combinations, HepG2 and THLE-2 cells, mice xenograft model

## Abstract

**Background**: Increasing evidence suggests that combinations of phytochemicals are more efficient than single components in the modulation of signaling pathways involved in cancer development. In this study, the impact of phenethyl isothiocyanate (PEITC), indole-3-carbinol (I3C), xanthohumol, (X), and resveratrol (RES) and their combinations on the activation and expression of Nrf2 and NF-κB in human hepatocytes and HCC cells were evaluated. **Methods**: THLE-2 and HepG2 cells were exposed to single phytochemicals and their combinations for 24 h. The activation of Nrf2 and NF-κB, expression of their target genes, and effect on cells survival were assessed. The tumor burden was evaluated in mice carrying xenografts. **Results**: All phytochemicals enhanced the activation and expression of Nrf2 and its target genes *SOD* and *NQO1* in HepG2 cells. The increased expression of *NQO1* (~90%) was associated with increased ROS generation. X + PEITC downregulated NF-κB activation reducing binding of its active subunits to DNA resulting in diminished *COX-2* expression. In contrast to single phytochemicals, X + PEITC induced apoptosis. Moderate reduction of tumor burden in mice carrying xenografts following X and PEITC or their combination was observed. **Conclusions**: Since Nrf2 is overexpressed in HCC its reduced activation together with diminished level of NF-κB by X + PEITC may be considered as a strategy to support conventional HCC therapy.

## 1. Introduction

Hepatocellular carcinoma (HCC) is the leading primary liver tumor and an example of inflammation-driven malignancy [1]. Several signaling pathways play a key role in the injury-inflammation-regeneration response. Among them, the classical NF-κB signaling is one of the most essential, activated especially during inflammation-related tumorigenesis. NF-κB usually is assigned in the form of p50–p65 heterodimer, representing the major Rel/NF-κB complex among cells. NF-κB in the latent state remains in the cytosol sequestered by its inhibitor, IκB protein. The presence of various stimulators leads to the activation of IκB kinase (IKK) responsible for phosphorylation of IκB. As a consequence, IKK undergoes proteasomal degradation, while NF-κB is translocated to the nucleus, where it stimulates the expression of genes such as cyclooxygenase-2 (*COX-2*) [2]. The overexpression of the latter is observed in human HCC and is linked with increased cell growth and invasiveness [1]. Furthermore, NF-κB transcription factor negatively interferes with the Nrf2 signaling pathway, which plays a protective role in hepatic inflammation [3]. Nrf2 is responsible for transcription genes encoding antioxidant and detoxifying enzymes essential in mitigating oxidative damage and reinstate the redox and metabolic homeostasis in the cells [4].

However, it is well-known that Nrf2 is extensively overexpressed in cancer cells, i.e., HCC, and might be a cause of the enhanced proliferation, invasion, and chemoresistance [5,6,7].

Nrf2, under normal conditions, binds in the cytosol to the Keap1 protein, which recruits ubiquitin ligase complex and leads to Nrf2 degradation in the proteasome. Upon stress conditions, a conformational change caused by the modification of key cysteine residues in Keap1 is observed. This modified form of Keap1 prevents Nrf2 ubiquitination and allows its translocation to the nucleus and initiation of target genes transcription [8]. This regulated way of activation of Nrf2, which takes place as a result of accelerated oxidative or electrophilic stress, is known as “canonical activation”. The autophagy-lysosome connection is another pathway that plays a crucial role in mediating oxidative stress response. Furthermore, there is an association between disturbed autophagy and activation of Nrf2. In this regard, autophagy blockage results in the accumulation of the autophagy adapter protein p62/SQSTM1 [9,10]. p62 is a multi-domain protein interacting with a host of protein targets. Thus, its increased concentration leads to the sequestration and loss of function of its multiple binding partners, e.g., Keap1 [11].

Therefore, the autophagosomal accumulation of p62 has been associated with the non-canonical activation of the Nrf2 signaling. Besides, several kinases, including ERK1/2 and GSK3β, may affect the Nrf2 activation [12].

Interestingly, non-canonical activation of Nrf2 can lead to both favorable or unfavorable impact on cancer development, and HCC is one of the most remarkable examples of harmful non-canonical activation of Nrf2. In this regard in HCC cell lines increased aggregates of p62 along with its phosphorylated form, as well as Keap1, responsible for the elevated levels of Nrf2, were shown [13]. Moreover, such changes were also found in HCC patient samples [14], proving that this mechanism of activation of Nrf2 might have a crucial role in the control of the progression of HCC. On the other hand, p62-related activation of Nrf2 has an essential contribution to the prevention of enhanced lipogenesis-dependent oxidative liver damage.

**Figure 1:**
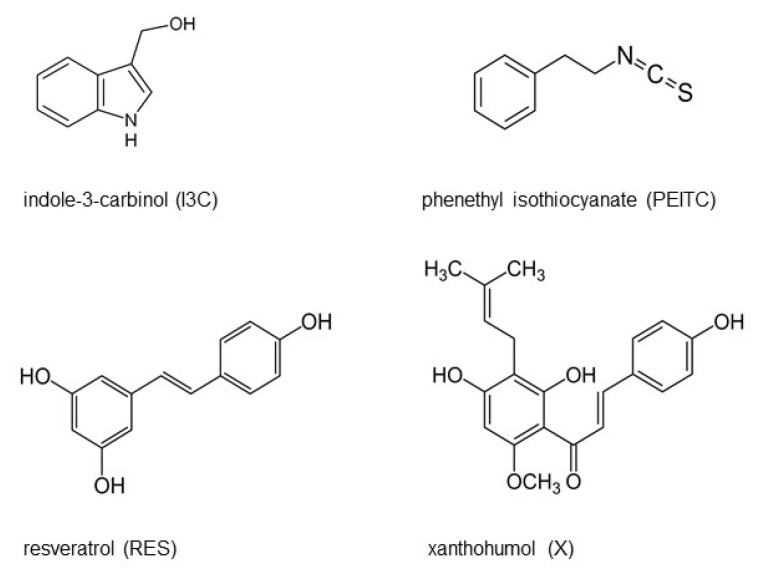
The tested phytochemicals—chemical structures.

Numerous studies indicated that naturally occurring phytochemicals (Figure 1), phenethyl isothiocyanate (PEITC), indole-3-carbinol (I3C) (products of glucosinolates degradation, present in cruciferous plants), xanthohumol (X, a chalcone, an ingredient of the hop plant (*Humulus lupulus* L.), and resveratrol (RES, a component of grape skin and seeds) exhibit anti-inflammatory, anti-carcinogenic, and anti-proliferative effects [15,16,17,18,19,20,21,22,23]. Our previous study indicated that I3C, PEITC, RES, and X decreased activation of NF-κB and enhanced activation of Nrf2 in pancreatic cancer cells (PANC-1, and Mia-Pa-Ca-1) [24,25]. Additionally, we observed that X alone induced the activation of Nrf2 in HepG2 [18]. Therefore, it seemed to be interesting to explore and compare the effect of these compounds in human-derived hepatoma HepG2 cells and normal immortalized human hepatocytes (THLE-2) particularly in the context of the different role of Nrf2 in cancer and normal cells.

Several recent studies have shown that using a combination of phytochemicals instead of single compounds may enhance their chemopreventive or therapeutic activities by reduction of excessive inflammation through modulation of several signaling pathways. Moreover, the combination of two or more phytochemicals may help to overcome the common problem of the disagreement between the high concentrations of most phytochemicals required in pre-clinical (in vitro and in vivo) studies and the low bioavailability of most phytochemicals after consuming relevant foods or supplements in humans [26,27].

Our recent study showed that the mixture X + PEITC was more efficient than single phytochemicals in decreasing NF-κB and in enhancement of activation and expression of Nrf2 in pancreatic cancer cells. Moreover, treatment of these cells with the X + PEITC led to their reduced proliferation [24].

Therefore, in this study we aimed to analyze the impact of X, PEITC, RES, and I3C and their mixtures on the expression and activation of canonical and non-canonical Nrf2 pathway in the human normal hepatocytes (line THLE-2) and hepatoma cells (line HepG2). Moreover, we evaluated the impact of those phytochemicals on the cell cycle, apoptosis, and interaction with NF-ĸB in HepG2 and their effect on tumor burden in mice xenografts.

## 2. Materials and Methods

### 2.1. Chemicals

I3C (purity ≥ 96%, CAS number 700-06-1), PEITC (purity 99%, CAS number 2257-09-2), X (purity ≥ 96%, CAS number 6754-58-1), RES (purity ≥ 99%, CAS number 501-36-0), dimethyl sulfoxide, Tris, solution of antibiotics (10^4^ U penicillin, 10 mg streptomycin, 25 μg amphotericin B), Dulbecco’s modified Eagle medium (DMEM), fetal bovine serum (FBS), trypsin, and topotecan were supplied by Sigma-Aldrich (St.Louis, MO, USA). Bronchial epithelial cell growth basal medium (BEGM) and Bullet Kit were purchased from Lonza/Clonetics Corporation (Basel, Switzerland). Primary antibodies against Nrf2 (sc-13032), SOD (sc-8637), NQO1 (sc-16464), GSK3β (sc-9166), p-GSK3β (sc-11757), Keap1 (sc-15246), NF-κB p50 (sc-114), NF-κB p65 (sc-7151), COX-2 (sc-376861), p62 (sc-28359), β-actin (sc-7210), lamin (sc-206800), and secondary alkaline phosphatase labeled antibodies were delivered by Santa Cruz Biotechnology (Dallas, TX, USA). Primary antibody against CAT (D4P7B) was obtained from Cell Signaling (Danvers, MA, USA). Horseradish peroxidase-labeled secondary antibodies were provided by Boster (Pleasanton, CA, USA). EURx (Gdańsk, Poland) provided the protein molecular weight marker.

### 2.2. Cell Culture and Viability Assay

The hepatocellular carcinoma cells, HepG2 (ATCC, Manassas, VA, USA), were grown in DMEM containing 10% FBS and 1% antibiotic solution and cultured at 37 °C, in humidified 5% CO_2_ atmosphere. Epithelial human liver cells-THLE-2 (ATCC, Manassas, VA, USA) were maintained in BEGM supplemented with Bullet Kit and 5 ng/mL EGF, 70 ng/mL phosphoethanolamine, and 10% FBS (37 °C, 5% CO_2_). After the 24h of initial incubation the cells (1 × 10^6^ cells per 100 mm culture dish) were treated with 5, 10, or 20 µM of tested compounds or their mixtures, incubated for additional 24 h, and harvested. Control cells were exposed to 0.1% DMSO. The doses of the phytochemicals were determined based on the MTT viability assay, which was performed according to the standard protocol. Briefly, HepG2 and THLE-2 cells were seeded (10^4^/well) in a 96-well plate and preincubated for 24 h. Subsequently, the cells were treated with X, RES, PEITC, I3C and their mixes at different concentrations increasing from 0.5 to 150 µM. After daily incubation, we rinsed the cells with PBS. The MTT salt (0.5 mg/mL) contained in the fresh medium was incubated for 4 h. In the last step, formazan crystals were dissolved in isopropanol containing HCl, and the absorbance was detected at 540 and 690 nm.

### 2.3. MUSE^®^ Cell Flow Cytometry Analysis

HepG2 cells (3 × 10^5^/well) were seeded in the 6-well plates and cultured for an initial 24 h. Then, studied phytochemicals were added and cells were subjected to 24 h incubation. Subsequently, cells were stained and assessed by flow cytometry on Muse^®^ Cell Analyzer, and data were generated using Muse^®^ 1.5 Analysis Software.

#### 2.3.1. Cell Cycle Distribution

The analysis of the cell cycle distribution is possible by propidium iodide staining and the specificity of its binding to DNA is improved by the addition of RNase A. Thus, the Muse^®^ Cell Cycle Kit (Merck, Darmstadt, Germany) was used in the research for the cell cycle analysis based on the manufacturer’s instructions. Topotecan (0.1 µM) treated cells acted as a positive control. After incubation cells were trypsinized, washed with PBS buffer, fixed in ice-cold 70% ethanol, and stored until staining at −20 °C. Before analysis fixed cells were washed with PBS buffer, stained, and subjected to 0.5 h incubation at room temperature in the dark.

#### 2.3.2. Proliferation

The Ki67 protein is expressed in proliferating cells. Its presence was evaluated by the Muse^®^ Ki67 Proliferation Kit (Merck, Darmstadt, Germany) according to the manufacturer’s instructions. Cells grown in a medium lacking FBS (starved) were served as a reference for the antiproliferative effect. After 24 h, cells were trypsinized and washed with PBS buffer. After fixation and exposure to permeabilization buffer, cells were subjected to incubation with the Muse^®^ Hu Ki67 Antibody for 0.5 h at room temperature in the dark.

#### 2.3.3. Apoptosis

Cells stained with the mixture of Annexin V (which identifies externalized phosphatidylserine in apoptotic cells) and 7-Aminoactinomycin (a marker of dead cells) were used in the determination of the percentage of cells undergoing early and late apoptosis. According to the manufacturer’s instructions, after incubation cells were trypsinized, resuspended in fresh medium, and stained for 20 min with the reagent from Muse^®^ Annexin V & Dead Cell Kit (Merck, Darmstadt, Germany). Topotecan (2 µM) treated cells served as a positive control of induced apoptosis.

#### 2.3.4. MAPK Pathway Activation

The ERK pathway is considered the canonical MAPK (mitogen-activated protein kinase) signaling pathway and can be evaluated as a reference of MAPK signaling activity. ERK1/2 phosphorylation is the marker of an active state of this pathway. The Muse^®^ MAPK Activation Dual Detection Kit (Merck, Germany) was used according to the manufacturer’s instructions. PMA (0.2 µg/mL) treated cells served as a positive control of the MAPK pathway activation. After incubation, cells were trypsinized and washed with PBS buffer. After fixation and exposure to permeabilization buffer, cells were incubated for 0.5 h at room temperature in the dark with a solution of two directly conjugated antibodies against ERK1/2: a phospho-specific (Thr202/Tyr204, Thr185/Tyr187) phycoerythrin and a total anti-ERK1/2-PECy5-conjugated antibody.

#### 2.3.5. Level of ROS

Solution of dihydroethidium (DHE) enables detection of reactive oxygen species (ROS) positive cells, as DHE reacts with superoxide radicals, generating fluorophores binding to DNA. The Muse^®^ Oxidative Stress Kit (Merck, Darmstadt, Germany) was used according to the manufacturer’s instructions for the quantitative measurement of superoxide radicals. After 24 h, cells were trypsinized, washed with PBS buffer, and resuspended in Assay Buffer containing Working Solution of Muse^®^ Oxidative Stress Reagent, and finally incubated for 0.5 h in 37 °C.

### 2.4. Preparation of RNA, Cytosolic, and Nuclear Extracts

The GeneMatrix Universal DNA/RNA/Protein Purification Kit (EURx, Gdańsk, Poland) was used to extract the total RNA, and the Nuclear/Cytosol Fractionation Kit (BioVision Research, Milpitas, CA, USA) was used to extract nuclear and cytosolic fractions. For further analysis, the samples were kept at −80 °C.

### 2.5. Quantitative Real-Time PCR

The Revert-Aid First Strand cDNA Synthesis kit (Fermentas, Burlington, ON, Canada) was used for total RNA reverse transcription followed by quantitative real-time PCR. Maxima SYBR Green Kit (Fermentas, Burlington, ON, Canada) and Chromo4 thermal cycler (BioRad Laboratories, Hercules, CA, USA) were applied. The protocol is presented in Table 1. The specificity of the products was verified by the melting curve. For data normalization, the estimated levels of *TATA box-binding protein* (*TBP*) and *porphobilinogen deaminase* (*PBDG*) were used. The primers were provided by oligo.pl (Warsaw, Poland) and are presented in Table 2.

### 2.6. Transcriptions Factors Binding Assays

To assess Nrf2 and NF-ΚB (p50 and p65 subunits) activation, enzymatic immunoassays with the Transcription Factor ELISA Assay Kit (TransAM™Nrf2/TransAM™NF-κB, Active Motif, Carlsbad, CA, USA) were used, based on the manufacturer’s protocol. Briefly, the proper consensus-site double-strand oligonucleotides (Nrf2-5′-GTCACAGTGACTCAGCAGAATCTG-3′; NF-κB-5′-GGGACTTTCC-3′) were immobilized on an ELISA plate and incubated for 1 h with the nuclear extracts. To recognize the binding of the activated subunits, DNAa-specific primary antibodies were added and detected with an HRP-conjugated secondary antibody. The colorimetric readout at λ = 450 nm was used to determine the amount of specific subunits, which correlated with the activated Nrf2 or NF-κB transcription factors.

### 2.7. Western Blotting

To determine the protein levels of NF-κB p50, NF-κB p65, Nrf2, COX-2, SOD, NQO1, CAT, GSK3β, p-GSK3β, Keap1, lamin, β-actin, p62 the immunoblot assays were performed. Nuclear (Nrf2, NF-κB p50, NF-κB p65, lamin) or cytosolic (Nrf2, Keap1, SOD, NQO1, CAT, p62, GSK-3β, P-GSK-3β, COX-2 and β-actin) fractions were separated on 10% and 12% SDS-PAGE gels and transferred to nitrocellulose membranes. Subsequently, skimmed milk (10%) was used as a blocking solution. After blocking, the proteins were incubated with dedicated primary antibodies. As an internal control lamin and β-actin were used. Alkaline phosphatase- and horseradish peroxidase-labeled anti-goat IgG and anti-rabbit IgG were applied as secondary antibodies. For alkaline phosphatase-labeled antibodies, bands were visualized in the staining reaction with AP Conjugate Substrate Kit (NBT/BCIP) (BioRad, Hercules, CA, USA). The products of reactions horseradish peroxidase-labeled antibodies with Clarity Western ECL Substrate (BioRad, Hercules, CA, USA) were visualized by chemiluminescence. The amount of the protein in each lane based on the intensity of the band was quantified by the Image Lab software (BioRad Laboratories, Hercules, CA, USA).

### 2.8. In Vivo Tumor Growth

Animal experiments were performed with the approval of the Poznan Local Ethics Committee for the Animal Experiments (date of approval: 5 April 2019; decision number 18/2019). Twelve -week-old male nude athymic BALB/c nu/nu mice were purchased from Charles River Laboratory (Sulzfeld, Germany). The animals were housed in individually ventilated cages at 12/12 h light/dark cycle and provided ad libitum with standard diet and water. HepG2 cells were stably transfected with a luciferase reporter gene using the following protocol Vector: pGL4.51 (Promega, Madison, WI, USA), Transfection reagent: Viafect (Promega, USA) Ratio DNA: Transfection Reagent- 1:4, incubation time with vector: 48 h. For selection: G418 at a dose of 5000 µg/mL was used, the cells were maintained in selection medium for two weeks in DMEM (+10% FBS, -P/S). The obtained clones were checked in the IVIS Spectrum (Caliper Life Sciences, Hopkinton, MA, USA) system using luciferin (GoldBio, St. Louis, MO, USA) (stock solution of 30 mg/mL) in PBS. HepG2 cells were collected from the flasks, counted and resuspended in concentration 8 × 10^6^ per 100 µL of cold PBS with Matrigel (30%) (Corning Life Science, Corning, NY, USA), and implanted subcutaneously into the right flank. The implantation of the xenograft was checked after five days. For this purpose, the luciferin (GoldBio, St. Louis, MO, USA) (150 mg/kg b.w.) was injected intraperitoneally. The animals were transferred from the anesthesia chamber to the Photon Imager instrument (BioSpace Lab, Nesles-la-Vallée, France) where they were kept under isoflurane-induced anesthesia. Then the luminescence was monitored in the selected region of interest until a plateau has been reached.

Once tumor xenografts reached ~50 mm^3^ (usually 12 days after implantation), the mice were assigned into four groups: (1) vehicle (ethanol 96%, PEG-400, and sterile sodium chloride 0.9%) treated control (*n* = 8); (2) X-treated group (*n* = 8); (3) PEITC-treated group (*n* = 8); and (4) X + PEITC-treated group (*n* = 8). The phytochemicals were administered intraperitoneally at the concentration of X 40 mg/kg b.w. and 15 mg/kg b.w. of PEITC or vehicle respectively at days 12th, 14th, and 17th. On day 18th after implantation, mice were sacrificed, and the tumor tissues were harvested.

### 2.9. The Assessment of the Type of Interaction between the Compounds

The combinatorial effects of the compounds on cell viability were evaluated by the analysis of the combination index (CI) using the CompuSyn 1.0 software (www.combosyn.com, accessed on 16 August 2021) [28]. The synergistic action of the compounds in combinations (defined as Fraction affected–Fa; values between 0 (no effect) and 1 (maximal effect)) was identified when CI ˂ 1.

### 2.10. Statistical Analysis

The data were analyzed by GraphPad Instat version 3.10. (GraphPad Software, San Diego, CA, USA). To assess the significance of the differences in the evaluated parameters one-way ANOVA with Dunnett’s post-hoc test was performed with the significance level of *p* < 0.05 and *p* < 0.01.

## 3. Results

### 3.1. Impact of X, PEITC, RES, I3C and Their Mixtures on the Cell Viability and Determination of the Type of Interactions between Phytochemicals

**Figure 2:**
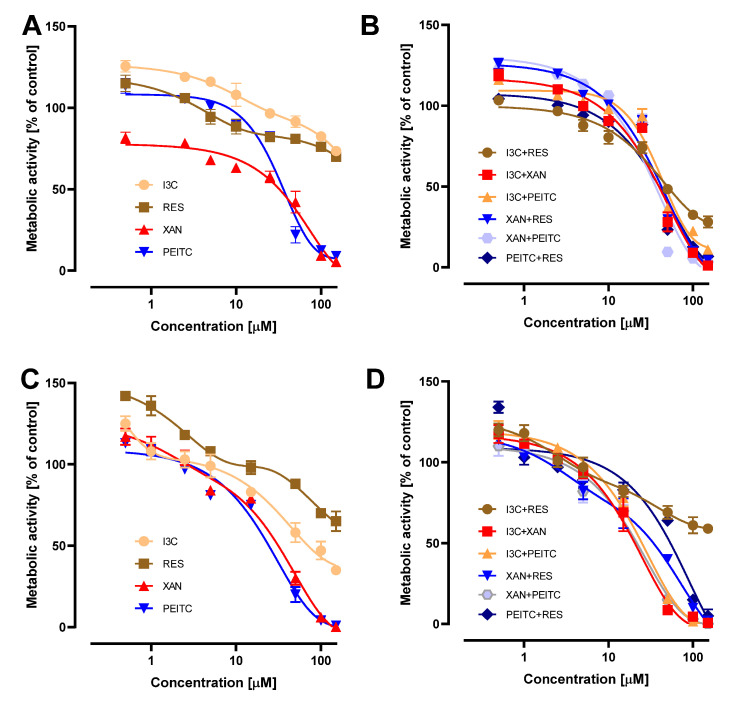
The impact of X, PEITC, RES, I3C, and their mixtures on cell viability of THLE-2 (panel **A**,**B**) and HepG2 (panel **C**,**D**) cells. Cell viability was estimated in comparison with vehicle control (100% viability). The values are shown as the mean ± SEM calculated from three independent experiments.

The effect of tested compounds and their combinations on the viability of THLE-2 and HepG2 cell lines was estimated based on the MTT assay within the concentration range of 0.5–150 µM (Figure 2). All compounds and their combinations diminished the viability of the cells in a dose-dependent manner. Table 3 presents respective IC_50_ values. PEITC showed the highest, while resveratrol had the lowest cytotoxicity. Slightly higher cytotoxicity toward hepatoma HepG2 cells in comparison with normal immortalized THLE-2 cells was observed. No significant difference was found between the cytotoxicity of single phytochemicals and applied mixtures.

Based on the estimation of safe concentration by cell viability assay, in further studies, we used the concentrations range from 5 to 20 µM for single compounds or their equimolar mixture.

**Figure 3:**
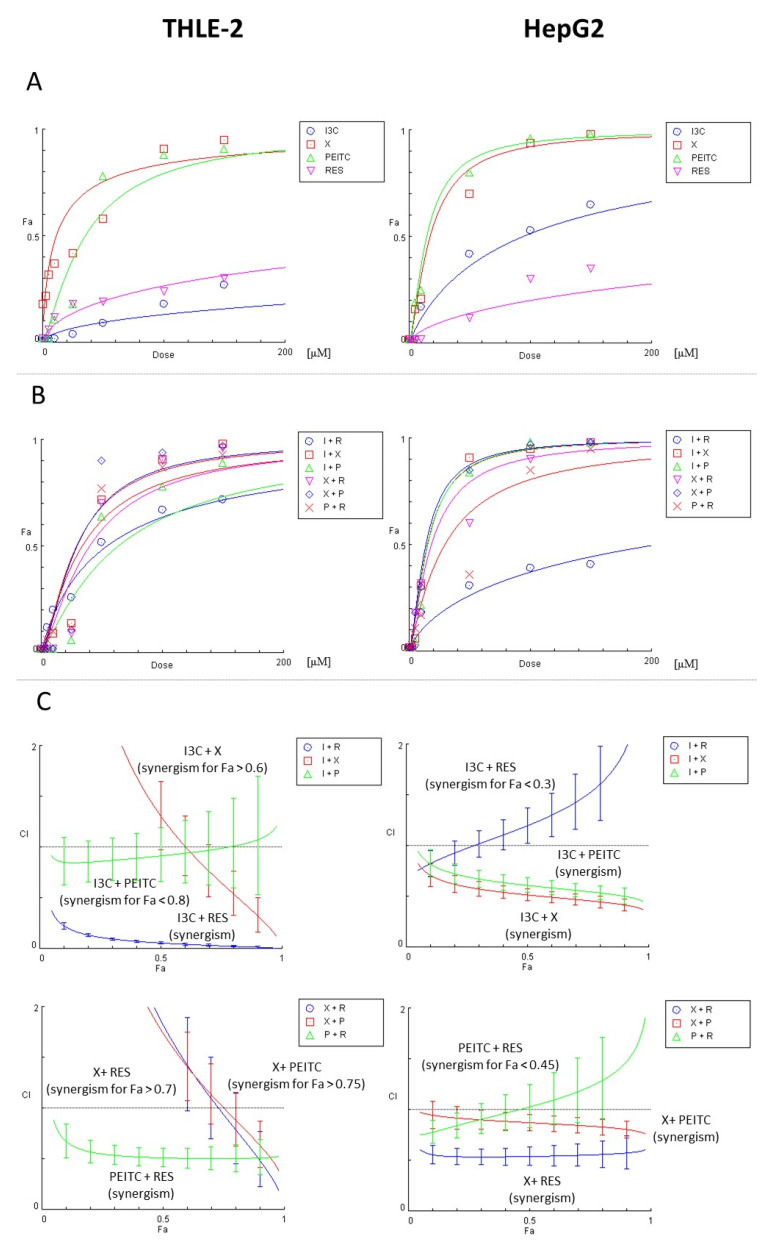
The results of the analysis of the combinatorial effects of the studied compounds on cell viability by MTT assay. THLE-2 and HepG2 cells were treated with increasing concentrations (0.5–150 µM) of the individual chemicals and their combinations for 24 h. Control cells were treated with vehicle (DMSO). Mean values from three independent experiments were used in calculations using CompuSyn software. Dose-effect curves for individual compounds (panel **A**) or their combinations (panel **B**) were generated. (Panel **C**) The evaluation of the combination index (CI) for the assessment of the synergism (CI < 1; the area below the dotted line in plots) between chemicals. Fa–Fraction affected: viability reduction from lack of effect (0) to maximal effect (1; viability = 0%).

In addition, we also evaluated the type of interaction between compounds in combinations by performing the combination index (CI) analysis in CompuSyn software (Figure 3). In THLE-2 epithelial human liver cells, synergistic reduction of viability was observed typically for I3C + RES and PEITC + RES combinations. Furthermore, in the majority of concentrations I3C + PEITC worked slightly synergistically (CI < 1). What is important, other mixtures presented synergy only in the high concentrations leading to significant reduction of viability: Fa > 0.6, Fa > 0.7, and Fa > 0.75 for I3C + X, X + RES, and X + PEITC, respectively.

On the contrary, for HepG2 hepatocellular carcinoma cells synergism was shown for I3C + PEITC, I3C + X, X + PEITC, and X + RES combinations. However, mixtures of I3C + RES and PEITC + RES seem to act mostly antagonistically (CI > 1), i.e., for Fa > 0.3 and Fa > 0.45, respectively. These combinations had a negative influence on cell viability, because both I3C + RES and PEITC + RES synergistically reduced the viability of THLE-2 cells, as well.

### 3.2. X, PEITC, RES, I3C, and Their Mixtures Affect the Activation of Nrf2

The Nrf2 activation was estimated based on the amount of Nrf2 isolated from the nuclei of THLE-2 or HepG2 cell lines in the oligonucleotide complex including the ARE consensus-binding site.

**Figure 4:**
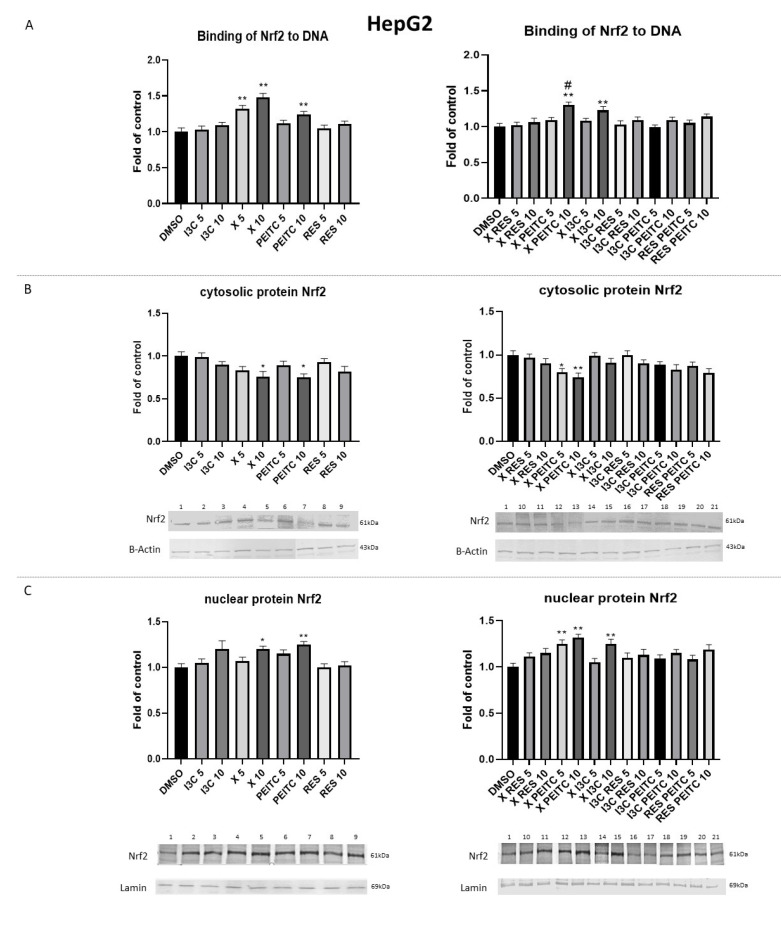
The impact of X, PEITC, RES, I3C, and their mixtures on the activation of Nrf2 in HepG2 cells. Panel (**A**) Activation of Nrf2 was estimated in terms of the amount of Nrf2 contained in the DNA-binding complexes extracted from the nuclear fraction. Panel (**B**) represents the immunoblots for the analysis of the cytosolic and panel (**C**) of the nuclear level of Nrf2 protein. The values are shown as the mean ± SEM calculated from three independent experiments (a fold of control) and determined by one-way ANOVA, post-hoc Dunnet test (* *p* < 0.05; ** *p* < 0.01). (#) above the bar indicates statistically significant differences between X + PEITC and X or PEITC group, *p* < 0.05 and *p* < 0.01. Lane 1, cells treated with DMSO; lane 2, I3C 5 µM; lane 3, I3C 10 µM; lane 4, X 5 µM; lane 5, X 10 µM; lane 6, PEITC 5 µM; lane 7, PEITC 10 µM; lane 8, RES 5 µM; lane 9 RES 10 µM; lane 10, X + RES 5 µM; lane 11, X + RES 10 µM; lane 12, X + PEITC 5 µM; lane 13, X + PEITC 10 µM; lane 14, X + I3C 5 µM; lane 15, X + I3C 10 µM; lane 16, I3C + RES 5 µM; lane 17, I3C + RES 10 µM; lane 18, I3C + PEITC 5 µM; lane 19, I3C + PEITC 10 µM; lane 20, RES + PEITC 5 µM; lane 21, RES + PEITC 10 µM.

X (by 32–48% at both tested concentrations-5µM and 10µM) and PEITC (by 24% at a higher concentration-10 µM) compared to untreated HepG2 cells significantly increased binding to the ARE consensus (Figure 4A). Treatment of HepG2 cells with their combination and the mixture of X and I3C at 10µM concentration increased Nrf2 binding only by ~25% (Figure 4A). The translocation of Nrf2 from the cytosol to the nucleus is required for its activation. In concert with increased binding to the ARE sequence, an increase of Nrf2 nuclear protein level (Figure 4C) was observed as a result of treatment with X and PEITC and their combination with a concomitant decrease of cytosolic protein (Figure 4B). The combination of X and I3C at a higher concentration also enhanced the Nrf2 nuclear protein level by ~25% in HepG2 cells.

**Figure 5:**
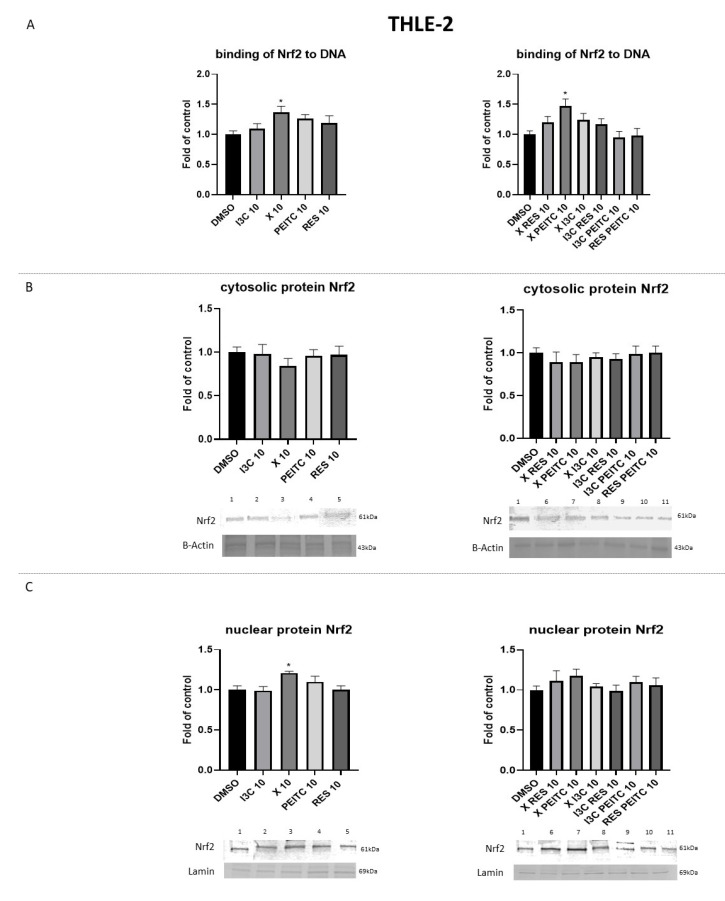
The impact of X, PEITC, RES, I3C, and their mixtures on the activation of Nrf2 in THLE-2 cells. Panel (**A**) The activation of Nrf2 was estimated in terms of the amount of Nrf2 contained in the DNA-binding complexes extracted from the nuclear fraction. Panel (**B**) presents exemplary immunoblots for the analysis of the cytosolic and panel (**C**) of the nuclear level of Nrf2 protein. The values are shown as the mean ± SEM calculated from three independent experiments (a fold of control) and determined by one-way ANOVA, post-hoc Dunnet test (* *p* < 0.05). Lane 1, cells treated with DMSO; lane 2, I3C 10 µM; lane 3, X 10 µM; lane 4, PEITC 10 µM; lane 5, RES 10 µM; lane 6, X + RES 10 µM; lane 7, X + PEITC 10 µM; lane 8, X + I3C 10 µM; lane 9, I3C + RES 10 µM; lane 10, I3C + PEITC 10 µM; lane 11, RES + PEITC 10 µM.

In THLE-2 cells, significant changes in the nuclear level of Nrf2 (Figure 5C) and binding to DNA were observed only in X and X + PEITC (Figure 5A).

### 3.3. X, PEITC, RES, I3C, and Their Mixtures Affect the Expression of Nrf2 Target Genes

**Figure 6:**
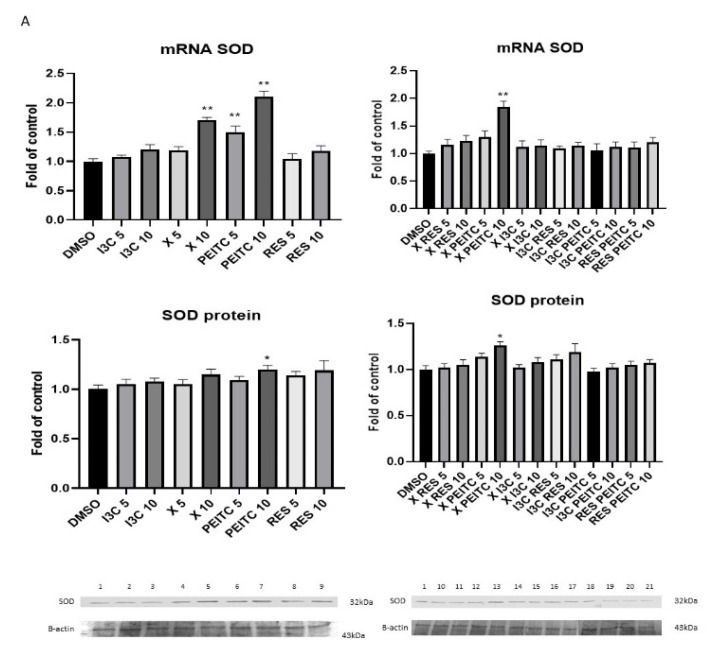

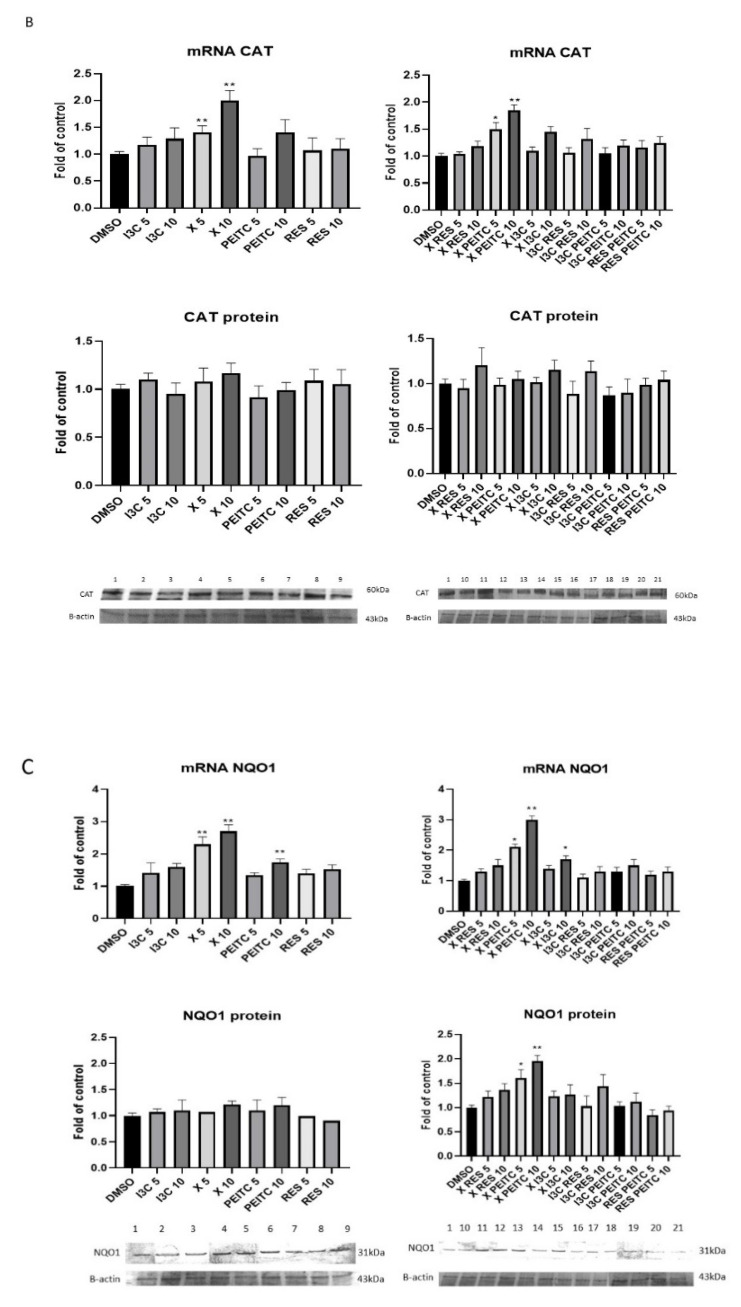
The impact of X, PEITC, RES, I3C, and their mixtures on the expression of Nrf2 target genes in HepG2 cells. SOD (panel **A**), CAT (panel **B**), and NQO1 (panel **C**) expression was calculated as mRNA level in comparison with control cells. Exemplary immunoblots for the analysis of protein level of SOD (panel **A**), CAT (panel **B**), and NQO1 (panel **C**). Results of the Western blot analysis are estimated as protein level in comparison with vehicle control. The values are shown as the mean ± SEM calculated from three independent experiments (a fold of control) and determined by one-way ANOVA, post-hoc Dunnet test (* *p* < 0.05; ** *p* < 0.01). Lane 1, cells treated with DMSO; lane 2, I3C 5 µM; lane 3, I3C 10 µM; lane 4, X 5 µM; lane 5, X 10 µM; lane 6, PEITC 5 µM; lane 7, PEITC 10 µM; lane 8, RES 5 µM; lane 9 RES 10 µM; lane 10, X + RES 5 µM; lane 11, X + RES 10 µM; lane 12, X + PEITC 5 µM; lane 13, X + PEITC 10 µM; lane 14, X + I3C 5 µM; lane 15, X + I3C 10 µM; lane 16, I3C + RES 5 µM; lane 17, I3C + RES 10 µM; lane 18, I3C + PEITC 5 µM; lane 19, I3C + PEITC 10 µM; lane 20, RES + PEITC 5 µM; lane 21, RES + PEITC 10 µM.

To estimate whether the activation of Nrf2 induced the expression of genes regulated by this transcription factor, namely *CAT*, *NQO1*, and *SOD*, the levels of their transcript and protein were assessed (Figure 6). The tested phytochemicals enhanced the expression of *CAT, NQO1*, and *SOD* at the transcripts levels in HepG2 cells. The strongest inducers of *CAT*, *NQO1*, and *SOD* were X and PEITC and their combination.

The enhanced expression was correlated with the elevated protein level in the case of SOD and NQO1 in the cells treated with PEITC and its combination with X resulting in ~20% and by ~70–96% increase, respectively.

### 3.4. X, PEITC, RES, I3C, and Their Mixtures Affect the Expression of Nrf2, Keap1, and p62

Figure 6 presents the effect of tested phytochemicals on Nrf2 gene transcript; Keap1 and p62 mRNA and protein levels.

**Figure 7:**
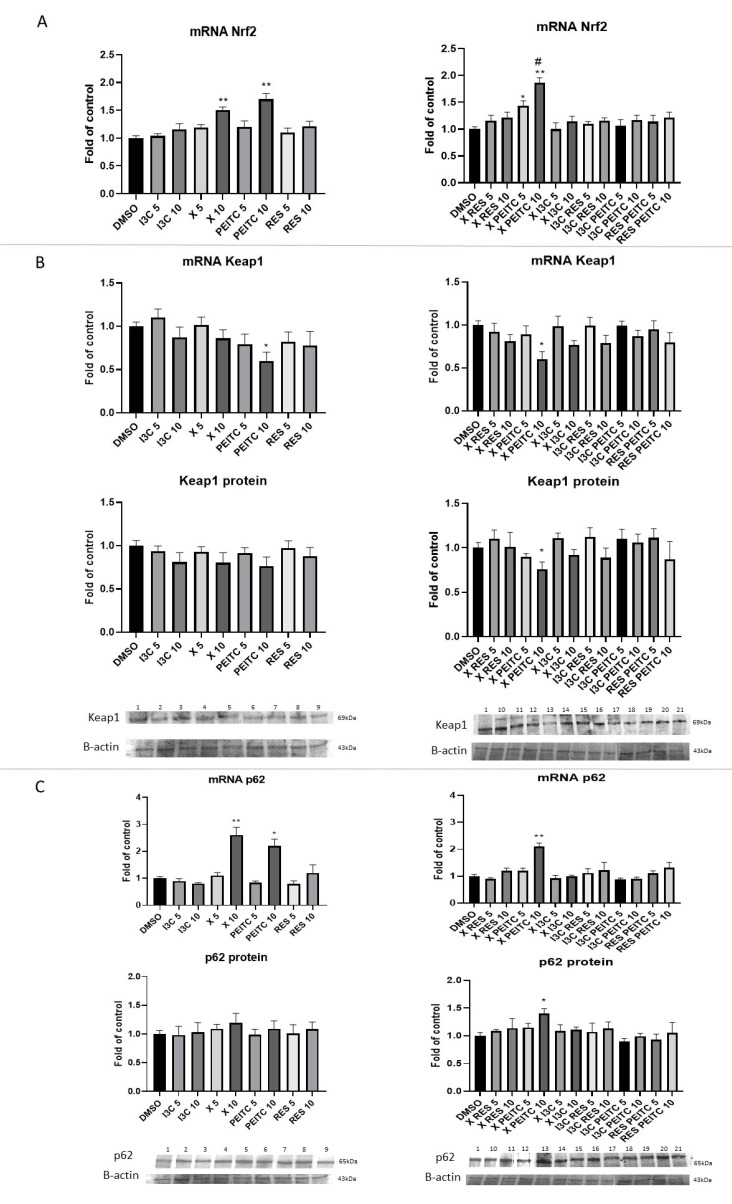
The impact of X, PEITC, RES, I3C, and their mixtures on the expression of Nrf2, Keap1, and p62 in HepG2 cells. Nrf2 (panel **A**), Keap1 (panel **B**), and p62 (panel **C**) expression was calculated as mRNA level in comparison with control cells. Exemplary immunoblots for the analysis of levels of Keap1 (panel **B**) and p62 (panel **C**) protein. Results of the Western blot analysis are estimated as protein level in comparison with vehicle control. The values are shown as the mean ± SEM calculated from three independent experiments (a fold of control) and determined by one-way ANOVA, post-hoc Dunnet test (* *p* < 0.05; ** *p* < 0.01). The asterisk (#) above the bar indicates statistically significant differences between X +PEITC and X or PEITC group, *p* < 0.05 and *p* < 0.01. Lane 1, cells treated with DMSO; lane 2, I3C 5 µM; lane 3, I3C 10 µM; lane 4, X 5 µM; lane 5, X 10 µM; lane 6, PEITC 5 µM; lane 7, PEITC 10 µM; lane 8, RES 5 µM; lane 9 RES 10 µM; lane 10, X + RES 5 µM; lane 11, X + RES 10 µM; lane 12, X + PEITC 5 µM; lane 13, X + PEITC 10 µM; lane 14, X + I3C 5 µM; lane 15, X + I3C 10 µM; lane 16, I3C + RES 5 µM; lane 17, I3C + RES 10 µM; lane 18, I3C + PEITC 5 µM; lane 19, I3C + PEITC 10 µM; lane 20, RES + PEITC 5 µM; lane 21, RES + PEITC 10 µM.

Treatment with X and PEITC at 10 µM concentration enhanced the transcription of *Nrf2* by ~50% and 70%, respectively. Their combination enhanced the effect of PETIC increasing *Nrf2* mRNA level by ~87% at the same concentration in comparison with the control (Figure 7A).

In order to partly explain the mechanism of Nrf2 activation, the effect of tested phytochemicals and their mixtures on *Keap1* and *p62* genes expression was assessed (Figure 7B,C). *Keap1* expression was affected only as a result of treatment with PEITC and its mixture with X, which reduced both Keap1 transcript and protein level by ~40% and ~30%, respectively.

X and PEITC and to more extent their combination at 10 µM significantly increased p62 transcript level by 160% and 120%, 110%, respectively. However, at the protein level increased expression of p62 was confirmed only in the case of the combination of X and PEITC.

### 3.5. X, PEITC, RES, I3C and Their Mixtures Affect GSK-3β Expression and Phosphorylation and ERK1/2 Phosphorylation

Besides p62, Keap1′s independent mechanism of Nrf2 activation and the regulation of *Nrf2*-ARE- mediated gene expression may involve also the activation of kinases GSK-3β and the ERK.

**Figure 8:**
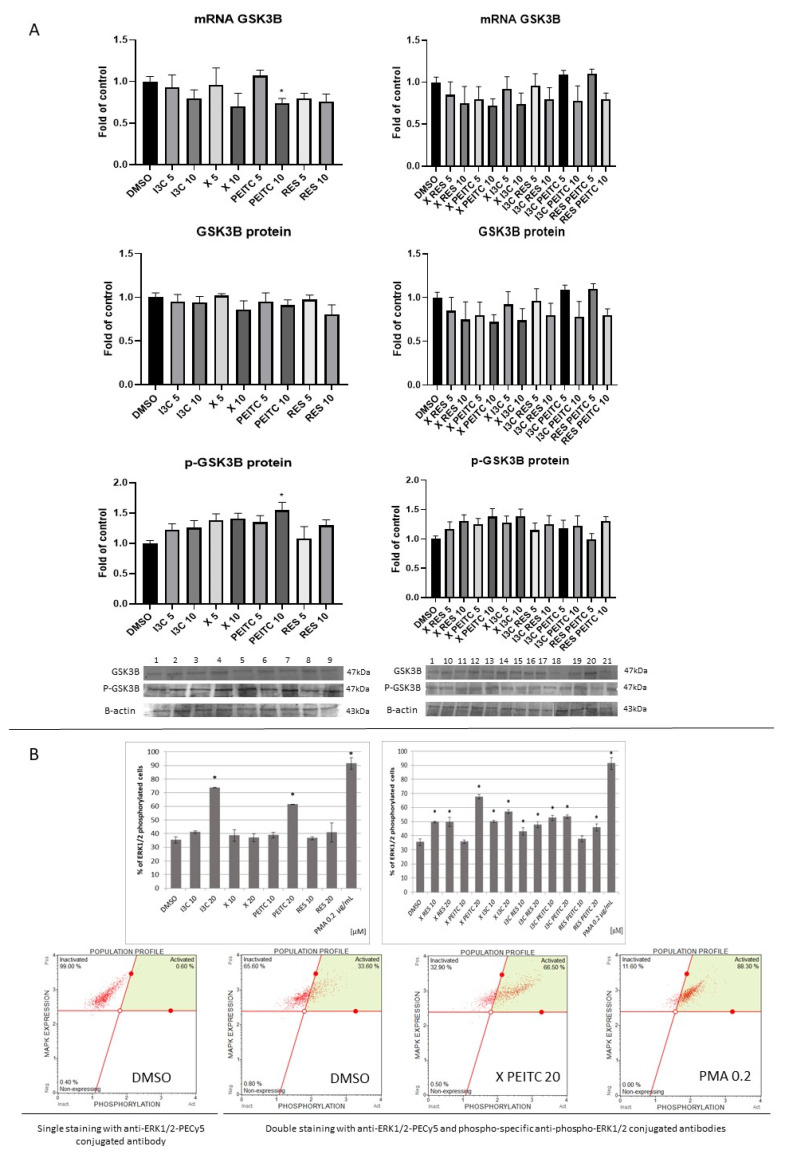
The impact of X, PEITC, RES, I3C, and their mixtures on GSK-3β expression and phosphorylation and ERK1/2 phosphorylation in HepG2 cells. Panel (**A**) GSK-3β expression was estimated as mRNA level comparing to control cells (a fold of control). Exemplary immunoblots are presented for GSK-3β and p-GSK-3β protein level. Western blot results are estimated as protein levels comparing to vehicle control (a fold of control). Panel (**B**) The impact of X, PEITC, RES, I3C, and their equimolar mixtures on ERK1/2 phosphorylation in HepG2 cells. The phosphorylation was measured by the flow cytometric analysis after double staining of cells with anti-ERK1/2-PECy5 and phospho-specific anti-phospho-ERK1/2 conjugated antibodies. PMA (para-methoxyamphetamine) was used as a positive control of ERK1/2 phosphorylation. The values are shown as the mean ± SEM calculated from three independent experiments (a fold of control) and determined by one-way ANOVA, post-hoc Dunnet test (* *p* < 0.05). Lane 1, cells treated with DMSO; lane 2, I3C 5 µM; lane 3, I3C 10 µM; lane 4, X 5 µM; lane 5, X 10 µM; lane 6, PEITC 5 µM; lane 7, PEITC 10 µM; lane 8, RES 5 µM; lane 9 RES 10 µM; lane 10, X + RES 5 µM; lane 11, X + RES 10 µM; lane 12, X + PEITC 5 µM; lane 13, X + PEITC 10 µM; lane 14, X + I3C 5 µM; lane 15, X + I3C 10 µM; lane 16, I3C + RES 5 µM; lane 17, I3C + RES 10 µM; lane 18, I3C + PEITC 5 µM; lane 19, I3C + PEITC 10 µM; lane 20, RES + PEITC 5 µM; lane 21, RES + PEITC 10 µM.

As it is shown in Figure 8A only PEITC treatment at the 10 µM concentration significantly reduced the *GSK-3β* transcript level but slightly increased its phosphorylated form (p-GSK-3β) protein level.

In contrast, treatment with I3C and PEITC, and all combinations increased the level of phosphorylated ERK1/2 (Figure 8B). The most effective were PEITC at the concentration of 20 µM and the mixture of X and PEITC increasing the level of its phosphorylated form by ~25% and ~33%, respectively. However, these values were lower than that obtained as a result of treatment with the reference compound, PMA (~57%).

### 3.6. X, PEITC, RES, I3C, and Their Mixtures Affect the NF-κB Activation and Expression of Its Active Subunits and COX-2 Gene

**Figure 9:**
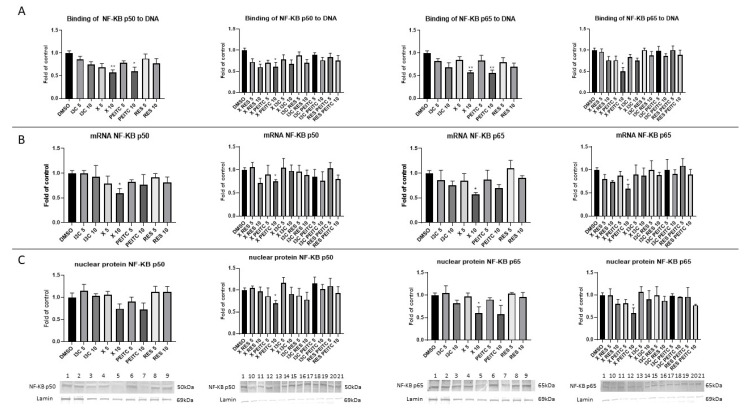
The impact of X, PEITC, RES, I3C, and their mixtures on NF-κB activation and expression of its active subunits in HepG2 cells. Panel (**A**) The activation of NF-κB p50 and p65 subunits was assessed in terms of the amount of NF-κB contained in the DNA-binding complexes extracted from the nuclear fraction. Panel (**B**) NF-κB p50 and p65 expression was calculated as mRNA level in comparison with vehicle control. Panel (**C**) Exemplary immunoblots are presented for the analysis of the NF-κB p50 and p65 protein levels. The values are shown as the mean ± SEM calculated from three independent experiments (a fold of control) and determined by one-way ANOVA, post-hoc Dunnet test (* *p* < 0.05; ** *p* < 0.01). Lane 1, cells treated with DMSO; lane 2, I3C 5 µM; lane 3, I3C 10 µM; lane 4, X 5 µM; lane 5, X 10 µM; lane 6, PEITC 5 µM; lane 7, PEITC 10 µM; lane 8, RES 5 µM; lane 9 RES 10 µM; lane 10, X + RES 5 µM; lane 11, X + RES 10 µM; lane 12, X + PEITC 5 µM; lane 13, X + PEITC 10 µM; lane 14, X + I3C 5 µM; lane 15, X + I3C 10 µM; lane 16, I3C + RES 5 µM; lane 17, I3C + RES 10 µM; lane 18, I3C + PEITC 5 µM; lane 19, I3C + PEITC 10 µM; lane 20, RES + PEITC 5 µM; lane 21, RES + PEITC 10 µM.

The NF-κB activation was measured based on the quantity of NF-κB active subunits p50 and p65 included in the DNA-binding nuclear complexes. As shown in Figure 9A, the amount of p50 in the DNA-binding complex in HepG2 cells was diminished by ~40% after treatment with X and PEITC and combinations of X and PEITC and X and RES at the 10 µM concentration. The quantity of p65 in the DNA-binding complex (Figure 9A) was reduced also by X and PEITC and their combination at the higher concentration (by ~43–50%). Similar changes were noted for p65 nuclear protein level (Figure 9C). The highest reduction of p50 nuclear protein level also was observed for mixtures of X and PEITC. At the 10 µM concentration X and its combination with PEITC reduced by 25–43% the transcript of both p50 and p65 subunit (Figure 9B).

**Figure 10:**
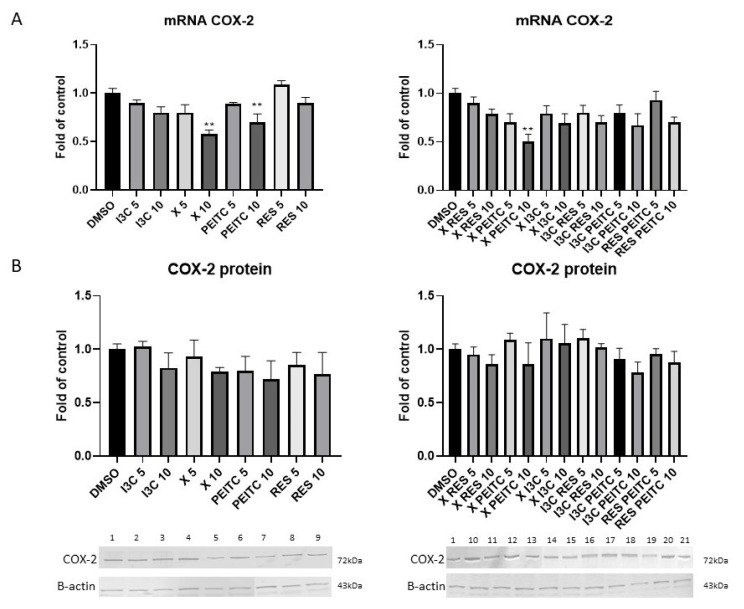
The impact of X, PEITC, RES, I3C, and their mixtures on the expression of COX-2 in HepG2 cells. Panel (**A**) COX-2 expression was calculated as mRNA level in comparison with control cells. Panel (**B**) Exemplary immunoblots are presented for the analysis of COX-2 protein level. Results of the Western blot analysis are expressed as protein level in comparison with vehicle control. The values are shown as the mean ± SEM calculated from three independent experiments (a fold of control) and determined by one-way ANOVA, post-hoc Dunnet test (** *p* < 0.01). Lane 1, cells treated with DMSO; lane 2, I3C 5 µM; lane 3, I3C 10 µM; lane 4, X 5 µM; lane 5, X 10 µM; lane 6, PEITC 5 µM; lane 7, PEITC 10 µM; lane 8, RES 5 µM; lane 9 RES 10 µM; lane 10, X + RES 5 µM; lane 11, X + RES 10 µM; lane 12, X + PEITC 5 µM; lane 13, X + PEITC 10 µM; lane 14, X + I3C 5 µM; lane 15, X + I3C 10 µM; lane 16, I3C + RES 5 µM; lane 17, I3C + RES 10 µM; lane 18, I3C + PEITC 5 µM; lane 19, I3C + PEITC 10 µM; lane 20, RES + PEITC 5 µM; lane 21, RES + PEITC 10 µM.

Inhibition of the activation of NF-κB correlated with a significant reduction in the expression of *COX-2* gene was noticed after incubation with X and PEITC and their combination at a higher concentration by 30–50% (Figure 10). The decreased expression did not correlate with analogous changes in the protein level of COX-2.

### 3.7. Impact of X, PEITC, RES, I3C, and Their Mixtures on Cell Cycle Distribution and Apoptosis Induction

**Figure 11:**
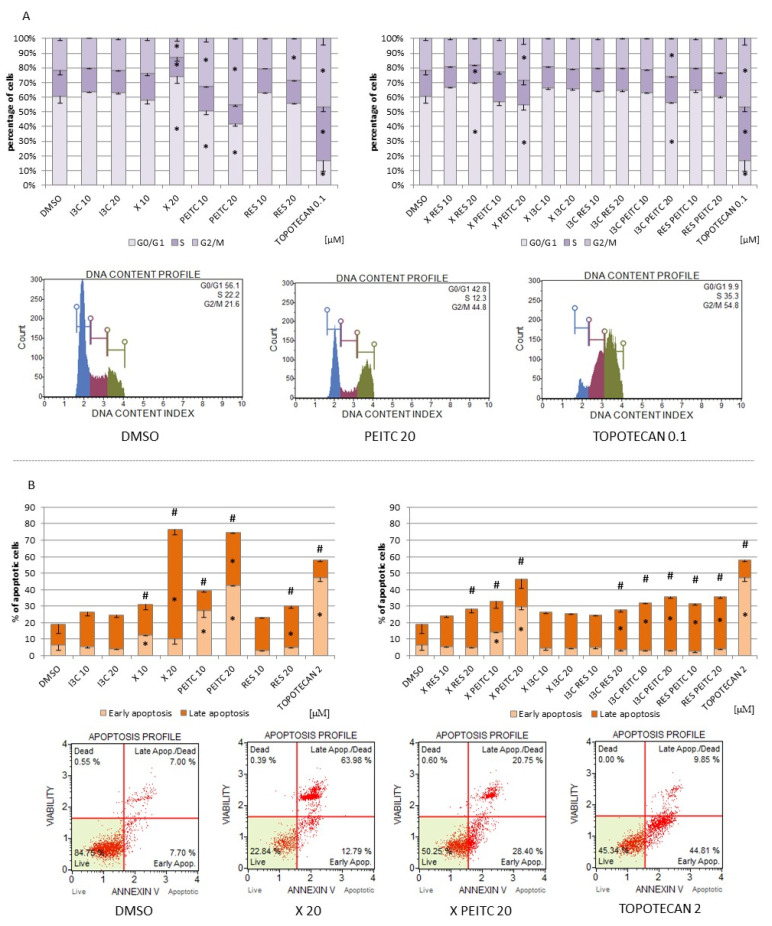
The impact of X, PEITC, RES, I3C, and their mixtures on cell cycle distribution and induction of apoptosis in HepG2 cells. Panel (**A**) The percentage of cells in G1/G0, S and G2/M phase analyzed by the flow cytometry after staining with propidium iodide and RNase A. Panel (**B**) The percentage of cells in the early and late stage of the apoptosis evaluated by the flow cytometry measurements based on fluorescence signal from Annexin V bound to phosphatidylserine externalized in apoptotic cells and a dead cell marker 7-AAD. Topotecan was used as a reference for cell cycle arrest (**A**) and pro-apoptotic activity (**B**). Exemplary plots are presented. Results were calculated from three separate experiments (mean ± SEM). (*) above the bar indicates statistically significant differences from the control group, while hashes (#) indicate statistically significant changes in the percentage of total apoptotic cells, *p* < 0.05.

Figure 11A shows the effect of single compounds and their combinations on cell cycle distribution. The differences in cell cycle distribution were observed in HepG2 cells treated with PEITC, with significant reduction of cells in G0/G1 and increase in G2/M, while X increased G0/G1 and decreased the percentage of cells in the G2/M phase. Furthermore, combination of X + RES increased the percentage of cells in G0/G1, while the treatment with the mixture of X + PEITC and I3C + PEITC elevated the percentage of cells in G2/M phase and reduced in phase G0/G1.

Cell distribution was changed in all phases after incubation with topotecan, a positive control, but this compound to a higher extent raised the quantity of cells in the G2/M phase.

The effect of the tested phytochemicals on the induction of apoptosis is presented in Figure 11B. All tested phytochemicals, except I3C, increased the number of apoptotic cells, but X and PEITC were the most effective apoptosis inducers (~55% increase of total apoptotic cells). The combination of the phytochemicals diminished the apoptotic effect of single compounds by ~27%.

### 3.8. Impact of X, PEITC, RES, I3C, and Their Mixtures on the Level of Reactive Oxygen Species and Cell Proliferation

**Figure 12:**
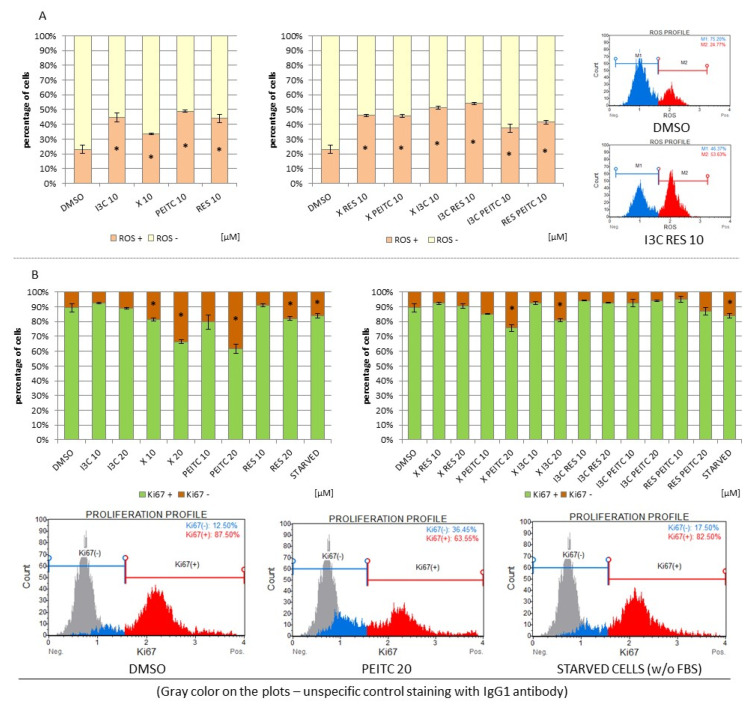
The impact of X, PEITC, RES, I3C, and their mixtures on the ROS production and on cell proliferation in HepG2 cells. Panel (**A**) The percentage of reactive oxygen species negative (ROS (−); M1) and positive (ROS (+); M2) cells based on dihydroethidium reaction with superoxide. The fluorescent signal was analyzed by flow cytometry. Exemplary plots are presented on the right side. Panel (**B**) The percentage of proliferating (Ki67 (+)) and non-proliferating (Ki67 (−)) cells were analyzed by flow cytometry. Starved cells (cultured without FBS) were acted as a reference to antiproliferative conditions. Exemplary plots are presented. Results were calculated from three separate experiments (mean ± SEM). (*) above the bar indicates statistically significant differences from the control group, *p* < 0.05.

As shown in Figure 12A all the tested compounds generated ROS. No significant differences between the effect of single phytochemicals and their mixtures were observed.

However, at the same time, an antiproliferative effect was found in the case of X and PEITC which exceeded that observed in starved cells (Figure 12B). The combination of these compounds as well as X + I3C also limited the HepG2 cells proliferation, but to a lesser extent than single compounds.

### 3.9. Antitumor Efficacy of PEITC, X and Their Combination in a Human Hepatic Tumor Xenograft Model

We evaluated the anti-cancer efficacy of the most active phytochemicals tested in this study in nude mice bearing HepG2 tumor xenografts.

**Figure 13:**
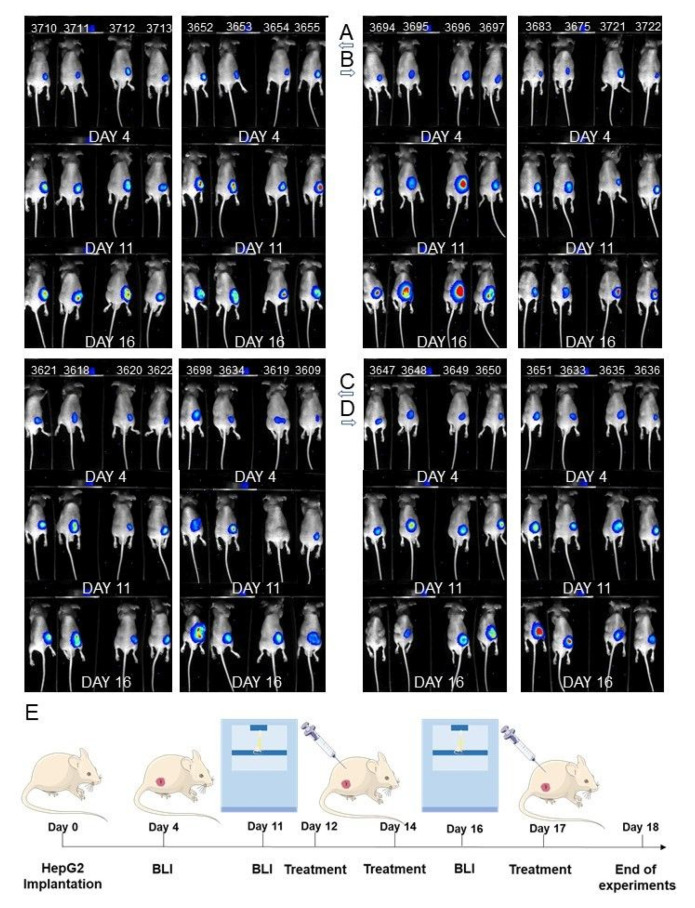
Effect of tested compounds on the tumor burden in BALB/c nude mice with HepG2 cancer xenografts. Control mice are shown in Panel (**A**), while the Xanthohumol-treated (40 mg/kg b.w.) group is presented in panel (**B**). The group treated with PEITC (15 mg/kg b.w.) on panel (**C**) and the group treated with Xanthohumol + PEITC on panel (**D**). The series of photographs show luminescence at days 4, 11, and 16, as is presented in panel (**E**). The luminescence signal measured in each mice (marked with ear tags’ number) is also shown in Figure 13.

**Figure 14:**
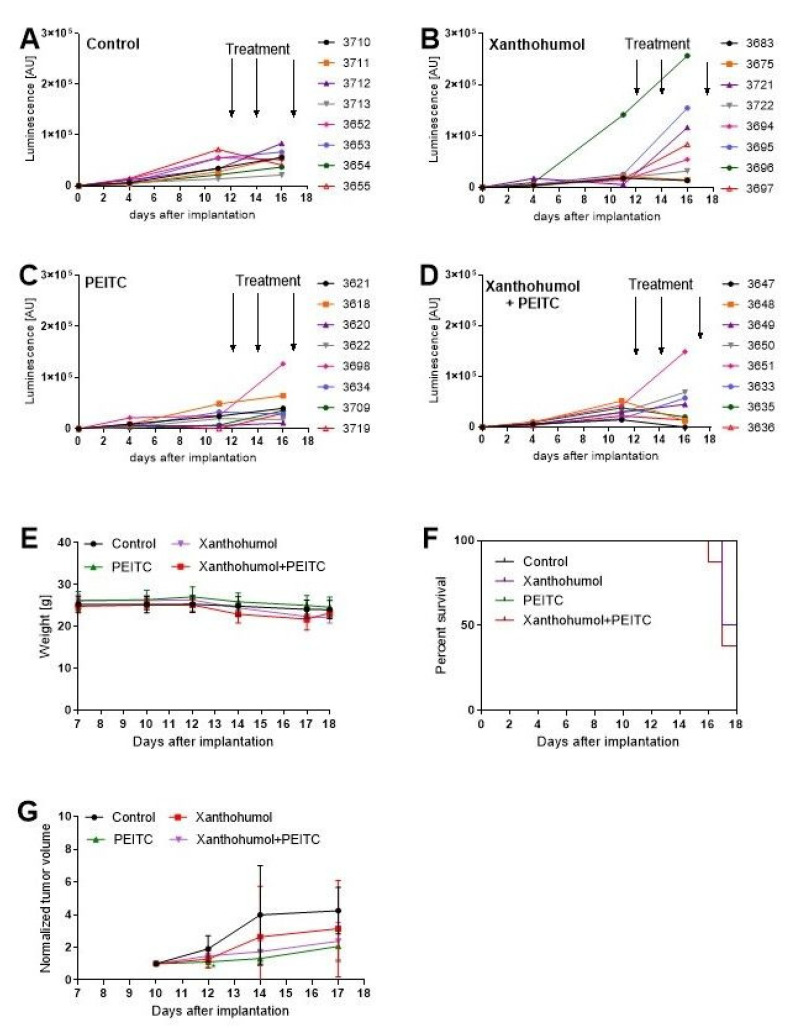
The in vivo efficacy and toxicity of single compounds and combination. Panels (**A**–**D**) present the luminescence of tumors, measured at day 4, 11, and 16 after implantation; panel (**E**) present the changes in mice weight during the experiments; Panel (**F**) presents the survival using the Kaplan–Meier curve; panel (**G**) presents the normalized tumor volume; tumor volume was measured using the formula V = 0.5 × W^2^ × L, where W and L mean width, and length, respectively. Tumor size was measured using a caliper. Statistical analysis was performed using GraphPad version 8.00 (GraphPad Software, San Diego, CA, USA). The data are shown as the means ± SD. The statistical significance was analyzed using one-way ANOVA with post-hoc Dunnett’s test. A *p*-value less than 0.05 was considered significant.

As shown in Figure 13 and Figure 14, both phytochemicals, at the concentrations selected for this experiment, showed the tendency to reduce tumor growth. However, although the differences between control and treated group of animals were visible, they could not be considered statistically significant. Similarly, the body weight in the phytochemicals-treated group of mice showed a reducing trend, but again the differences were not statistically significant in comparison with control.

## 4. Discussion

The objective of this study was to search for the possible synergism of phytochemicals, well-known for their chemopreventive activity, in the modulation of signaling pathways playing key roles in inflammation and cancer development in human hepatocellular carcinoma cells.

Initial evaluation of possible synergism of tested phytochemicals in combinations on the effect on cells viability, showed a synergetic reduction of cell viability of both cell lines, although for different mixtures. In HepG2 cell line, synergism has been confirmed for X + PEITC, I3C + PEITC, I3C + X, and X + RES combinations, on the contrary, in THLE-2 cell line synergistic reduction of viability was observed typically for I3C + RES and PEITC+ RES combinations.

Overall, the results of this study confirmed our and the other’s earlier observations on the ability of all tested phytochemicals, particularly X and PEITC to induce the Nrf2 signaling pathway and reduce the activation of NF-κB in HepG2 cells [18,29].

It has to be underlined that the most effective concentration of xanthohumol and phenethyl isothiocyanate i.e., 10 µM is equimolar of that consumed in beer and some vegetables, respectively [30,31].

Among the tested mixtures, the combination X + PEITC was the most efficient. However, in contrast to our recent findings in pancreatic cancer PANC-1 cells, their combination was equal or less efficient than the effect of single compounds at least in the activation of Nrf2 [24]. This difference may result from the specificity of these cells.

While the expression of Nrf2 is up-regulated in pancreatic cancer cell lines, HepG2 cells appear to exhibit average sensitivity to chemically induced ARE activation [32,33].

In this regard, X increased Nrf2 binding to target ARE sequence in HepG2 cells by almost 50%, while its combination only by 25%. Activation of Nrf2 resulted in increased expression of Nrf2 target genes, *SOD*, *NQO1,* and to a lesser extent *CAT*. Interestingly, *NQO1* expression was the most increased as a result of treatment with the combination of X and PEITC enhancing twice its protein expression in comparison with vehicle-treated control.

NQO1-mediated catalysis is widely regarded as beneficial, as it avoids an undesirable one-electron reduction that is directly associated with the radical formation and oxidative stress [34]. However, NQO1 may also mediate the generation of hydroquinones, which are labile and ultimately lead to the production of intracellular ROS. Such effect is especially prominent in cancer cells since these cells are reported to contain higher activities of NQO1 than non-cancerous cells [35]. Indeed, elevated levels of ROS were noted in our study as a result of treatment with the combination of X and PETIC, but also the other tested phytochemicals. Therefore, the possible mechanism of ROS formation as a result of hydroquinones generation is not fully supported.

Nrf2 activation may occur through the canonical and non-canonical pathways. The first one is linked with the degradation of Keap1 protein and subsequently its reduced level.

Such effect, as well as reduction of *Keap1* mRNA level, was observed as a result of treatment with PEITC and its mixture with X. However, p62 expression, the key player of non-canonical was also elevated, but only on mRNA level. The p62 protein level was also elevated but to a lesser extent, which suggests that tested phytochemicals do not affect its translation or post-translational modification.

The difference between mRNA levels and the corresponding protein levels may indicate that many mRNA molecules do not reach the translational machinery, probably because the translation mechanism is saturated in the conditions of enhanced transcription. Besides, p62 protein, the activation of protein kinases such as ERK, induces Nrf2 phosphorylation, which may stimulate the dissociation of Nrf2 from its repressor Keap1 and subsequent translocation into the nucleus [36]. The level of the active phosphorylated form of ERK was elevated as a result of treatment with I3C and PEITC and all mixtures, but basically, at a higher concentration such as 20 µM that was not applied in the other assays.

The phosphorylated GSK3β was only slightly increased or not affected, which correlates with relatively low activation of Nrf2 since phosphorylation of GSK3β leads to its inactivation with simultaneous activation of Nrf2 [12].

These results indicate that both mechanisms of Nrf2 activation i.e., canonical and non-canonical may be affected by the tested phytochemicals and their combinations.

The most significant effect of both X and PEITC applied as single compounds and in combination was noted in the activation of NF-κB. Particularly, the combination of these phytochemicals reduced binding to NF-κB specific sequence and increased the nuclear protein level of its p65 subunit, which is responsible for the initiation of the transcription process [37]. Since the disproportionate increase in the activated p65 and subsequent transactivation of effective molecules is an integral part of carcinogenesis, the NF-κB p65 is considered a pivotal target of chemopreventive and/or chemotherapeutic agents [38]. Therefore, the decreased level of NF-κB p65, followed by lower expression and protein level of COX-2, its target gene, as a result of treatment by the combination of X and PEITC and X alone might be considered as beneficial. Moreover, the increasing amount of evidence suggests that high levels of Nrf2 activity may promote cancer growth and increase chemoresistance. In this regard sustained Nrf2 activation has been observed in HCC and was thought to facilitate its progression and aggressiveness [39].

Therefore, relatively low induction of Nrf2 by this combination in HepG2 cells together with the reduced activation of NF-κB may qualify this mixture to support conventional HCC therapy.

NF-κB is generally regarded as anti-apoptotic, however, in particular contexts and especially in response to cellular stress NF-κB acts to promote apoptosis [40].

The reduced activation of NF-κB observed in our study as a result of treatment with the combination of X and PEITC did not affect the cell cycle distribution and induced apoptosis to a lesser extent than X alone. This effect only partly can be explained by ROS generation since increased levels of intracellular ROS were found after treatment with all evaluated phytochemicals alone and their combinations. In contrast to pancreatic cancer cells, the mixture of X and PEITC was also less efficient than its single components in the inhibition of HepG2 cells proliferation. Since both compounds i.e., X and PEITC are highly reactive it cannot be excluded that interaction of hydroxyl group of chalcone –X with ITC may lead to the formation of a product with lower pro-apoptotic and anti-proliferative activity. Further studies are required to verify this possibility. The moderate effect on HepG2 cells proliferation in vitro was confirmed in vivo in xenograft model mice transfected with HepG2 cells.

In summary, the present study confirmed our earlier observations that the combination of X and PEITC more efficiently than single components modulates the signaling pathways involved in inflammation, namely NF-κB. However, in contrast to pancreatic cancer cells, in hepatocellular carcinoma cells, this combination rather reduces the activation of the Nrf2-ARE pathway. Since the latter is often overexpressed in HCC this combination may be considered as a strategy to support conventional HCC therapy. Further studies, including a wider spectrum of concentrations and treatment protocols, are required to confirm this concept in vivo.

## Figures and Tables

**Table 1:** The protocol of qPCR.

	Temperature	Time
Enzyme activation	95 °C	5 min
50 cycles	95 °C	15 s
	56 °C	20 s
	72 °C	40 s
Final extension	72 °C	5 min

**Table 2:** The sequence of primers used in qPCR.

	Forward Primer	Reverse Primer
*PBGD*	5′TCAGATAGCATACAAGAGACC	5′TGGAATGTTACGAGCAGTG
*TBP*	5′GGCACCACTCCACTGTATC	5′GGGATTATATTCGGCGTTTCG
*Nrf2*	5′ATTGCTACTAATCAGGCTCAG	5′GTTTGGCTTCTGGACTTGG
*SOD*	5′CGACAGAAGGAAAGTAATG	5′TGGATAGAGGATTAAAGTGAGG
*CAT*	5′TGGACAAGTACAATGCTGAG	5′TTACACGGATGAACGCTAAG
*NQO1*	5′CAATTCAGAGTGGCATTC	5′GAAGTTTAGGTCAAAGAGG
*KEAP1*	5′ATGGGCGAGAAGTGTGTC	5′TCTGCTCAGCGAAGTTGG
*p62*	5′TCTGGGCATTGAAGTTGA	5′CTCTGTGCTGGAACTCTC
*GSK-3B*	5′ACCCAAATGTAAACTACCAAATG	5′TCCACGGTCTCCAGTATTAGC
*NF-κB p50*	5′ATCATCCACCTTCATTCTCAA	5′AATCCTCCACCACATCTTCC
*NF-κB p65*	5′CGCCTGTCCTTTCTCATC	5′ACCTCAATGTCCTCTTTCTG
*COX-2*	5′CCTGTGCCTGATGATTGC	5′CAGCCCGTTGGTGAAAGC

**Table 3:** The IC50 values for HepG2 and THLE-2 cell lines.

	HepG2		THLE-2	
	IC50	±SEM	IC50	±SEM
**I3C**	N/A		N/A	
**X**	34.2	2.3	37.0	3.0
**PEITC**	31.0	2.0	38.0	2.0
**RES**	N/A		N/A	
**X + I3C**	30.2	0.6	40.7	1.0
**X + PEITC**	26.5	1.6	37.5	1.2
**X + RES**	38.5	5.5	42.0	0.8
**I3C + PEITC**	32.2	1.0	44.5	0.8
**I3C + RES**	N/A		48.5	0.5
**RES + PEITC**	63.8	2.0	39.5	0.3

The IC50 values (*n* = 3) ±SEM (µM) were calculated based on the dose-response curves assessed by the MTT assay.

## Data Availability

The data is contained within this article.
